# Pilot Demonstration of Reclaiming Municipal Wastewater for Irrigation Using Electrodialysis Reversal: Effect of Operational Parameters on Water Quality

**DOI:** 10.3390/membranes11050333

**Published:** 2021-04-30

**Authors:** Xuesong Xu, Qun He, Guanyu Ma, Huiyao Wang, Nagamany Nirmalakhandan, Pei Xu

**Affiliations:** 1 Department of Civil Engineering, New Mexico State University, Las Cruces, NM 88003, USA; xuesong@nmsu.edu (X.X.); gyma@nmsu.edu (G.M.); huiyao@nmsu.edu (H.W.); nkhandan@nmsu.edu (N.N.); 2 Carollo Engineers, Phoenix, AZ 85034, USA; che@carollo.com

**Keywords:** electrodialysis, reclaimed water, desalination, ion-exchange membranes, ion permselectivity, irrigation

## Abstract

The modification of ion composition is important to meet product water quality requirements, such as adjusting the sodium adsorption ratio of reclaimed water for irrigation. Bench- and pilot-scale experiments were conducted using an electrodialysis reversal (EDR) system with Ionics normal grade ion-exchange membranes (CR67 and AR204) to treat the reclaimed water in the Scottsdale Water Campus, Arizona. The goal is to investigate the impact of operating conditions on improving reclaimed water quality for irrigation and stream flow augmentation. The desalting efficiency, expressed as electrical conductivity (EC) reduction, was highly comparable at the same current density between the bench- and pilot-scale EDR systems, proportional to the ratio of residence time in the electrodialysis stack. The salt flux was primarily affected by the current density independent of flow rate, which is associated with linear velocity, boundary layer condition, and residence time. Monovalent-selectivity in terms of equivalent removal of divalent ions (Ca^2+^, Mg^2+^, and SO_4_^2−^) over monovalent ions (Na^+^, Cl^−^) was dominantly affected by both current density and water recovery. The techno-economic modeling indicated that EDR treatment of reclaimed water is more cost-effective than the existing ultrafiltration/reverse osmosis (UF/RO) process in terms of unit operation and maintenance cost and total life cycle cost. The EDR system could achieve 92–93% overall water recovery compared to 88% water recovery of the UF/RO system. In summary, electrodialysis is demonstrated as a technically feasible and cost viable alternative to treat reclaimed water for irrigation and streamflow augmentation.

## 1. Introduction

The development of alternative water supplies such as municipal wastewater has been a viable solution to address the challenges of water scarcity and impaired water quality that have influenced human activities and the environment [1,2,3]. Reclaimed water has been used for irrigation of turf and crops [4,5], groundwater recharge [6,7], potable reuse [8], cooling water for power plants [9], and other municipal and industrial applications [10]. The total dissolved solids (TDS) in such reclaimed water sources (usually above 1000 mg/L) and elevated concentrations of specific ions (e.g., sodium, calcium, chloride, nitrate, and sulfate) can adversely impact potential beneficial use [11,12]. For instance, the use of reclaimed water containing high sodium concentration for agricultural and golf course irrigation would degrade soil condition [13,14], while the discharge of high chloride water may jeopardize surface water discharge permit compliance due to failure of the Whole Effluent Toxicity (WET) test [15].

Reverse osmosis (RO) is currently the primary membrane separation process for water desalination and reuse [16,17]. However, the application of RO is hindered by high energy demand, membrane scaling and fouling, and limited options for concentrate disposal [18]. Electrodialysis or electrodialysis reversal (EDR) that utilizes an electrical potential difference between the electrodes and ion-exchange membranes (IEMs) to remove salt ions provides an alternative to desalinate brackish water and wastewater [19,20]. The tendency of membrane scaling and fouling in electrodialysis can be significantly reduced because of paralleling water flow paths along the IEM surface (not passing through membranes) and relatively loose scaling/fouling layer due to low hydraulic pressure applied [21]. EDR enables the same electrochemical separation process as electrodialysis, except periodic reversal of the electrical polarity of the electrodes to inhibit deposition of potential scaling/fouling on the IEM surface [22,23]. In addition, electrodialysis barely rejects neutral species such as silica and organic matter, thereby further reducing the fouling and scaling propensity. Unlike RO that produces high purity permeate and requires post-treatment and stabilization to generate final product water, electrodialysis can flexibly tailor the desalination level and control ion composition in product water by adjusting operating parameters such as applied voltage, flow rate, and staging of the electrodialysis stacks. The electrodialysis process can directly produce product water with the desired water quality, thus avoiding the complexity of stabilizing the product water after desalination [24,25,26]. Therefore, electrodialysis could be a more cost-effective and energy-saving process than RO for desalination of impaired water sources for non-potable water reuse applications.

Electrodialysis has been a commercial technology for decades, primarily for groundwater desalination. There is an increasing interest in electrodialysis for various applications including irrigation because of its selective separation capacity. More investigation of wastewater treatment using electrodialysis is needed to explore the transport behaviors of ions in multi-component solutions due to the complex interactions of ions in the solutions and competing transport of different ions through IEMs simultaneously [27]. Currently, most electrodialysis studies in treating reclaimed water were conducted in laboratory-scale systems with synthetic or real solutions [28,29,30,31,32]. It is critical to treat wastewater at scale-up electrodialysis systems for evaluating the feasibility and economic viability through long-term near full-scale operations, and bridging bench- and pilot-scale experiments to predicting commercial implementation [33,34].

In addition, the impacts of operational parameters need to be thoroughly analyzed at large-scale electrodialysis system for system design and operation. The applied voltage and current density (Equation (3) in Supplemental Information) are of importance for the overall performance of electrodialysis system [35]. The flow rate, associated with linear velocity, is also a key operating parameter to determine overall desalination and energy efficiencies because it affects the degree of mixing and controls the residence time of the desalting water inside the electrodialysis stacks [36,37]. Therefore, the net influence of flow rate on the salt transfer rate merits further investigation for reclaiming wastewater. As scaling can pose a challenge for treating wastewater using electrodialysis at high water recovery [38,39], the impact of hydraulic water recovery is also worth being evaluated under various operating conditions.

This study investigated the application of electrodialysis to treat reclaimed water for golf course irrigation and instream flow augmentation. A bench-scale electrodialysis unit and a 2 electrical stage pilot-scale EDR system manufactured by the Suez Water Technologies & Solutions were operated continuously at an advanced wastewater treatment facility in Scottsdale, Arizona, USA, to investigate the technical feasibility and economic viability of the electrodialysis process for treating reclaimed water under different operating conditions. The reclaimed water needs to meet a sodium goal of 110 mg/L for golf course irrigation and a chloride goal of 150 mg/L to reduce the risks of WET failure for instream flow augmentation. The City of Scottsdale utilizes ultrafiltration (UF) as pretreatment and subsequent RO to desalinate a partial stream of the reclaimed water and blend it with the remaining stream. This approach requires subsequent blending and stabilization for final product water and generates a RO concentrate that requires proper disposal.

The present study aims to evaluate the use of EDR as an alternative to UF/RO for treating the reclaimed water for non-potable water reuse, e.g., irrigation and in-stream augmentation. The main objectives of the study include the following: (1) assess the sodium and chloride removal behavior in treating the reclaimed water to meet the target water quality requirements for golf course irrigation and surface water discharge; (2) evaluate the impacts of pilot-scale EDR configuration (e.g., number of staging and cell pairs) and operating conditions (e.g., applied voltage, flow rate, and water recovery) on desalination performance and ion composition of the product water; (3) compare the desalting performance between bench- and pilot-scale electrodialysis for system scale-up and optimization; and (4) conduct a techno-economic analysis of the EDR process in comparison to the existing UF/RO system.

## 2. Materials and Methods

### 2.1. Water Quality and Analysis

The bench- and pilot-scale experiments were conducted at the Scottsdale Water Campus, Arizona, treating the denitrified tertiary effluent from the wastewater treatment plant (WWTP). During the testing period, the TDS concentration of the reclaimed water was 1131 ± 34 mg/L with an electrical conductivity of 1812 ± 61 µS/cm. The major ions in the reclaimed water included Na^+^ (246 ± 30 mg/L), Ca^2+^ (85 ± 8 mg/L), Mg^2+^ (29 ± 1.4 mg/L), K^+^ (23 ± 1.2 mg/L), Cl^−^ (350 ± 18 mg/L), SO_4_^2−^ (253 ± 32 mg/L), and SiO_2_ (12.5 ± 1.1 mg/L). The relatively high concentrations of sodium and chloride make the reclaimed water unsuitable for beneficial reuse, specifically for irrigation in this case. The concentrations of other minor ions such as arsenic, phosphate, iron, manganese, nitrite, nitrate, bromide, and fluoride were negligible to impact the desalination performance. The pH of the reclaimed water was 7.4 ± 0.1, and the alkalinity was 155 ± 20 mg/L as CaCO_3_. The total organic carbon (TOC) concentration in the reclaimed water was 7.7 ± 0.15 mg/L.

The electrical conductivity and pH of the water samples were measured using a conductivity and pH meter (Model 431-61, Cole-Parmer, Vernon Hills, IL, USA). The TOC was determined by a carbon analyzer (Shimadzu TOC-L, Kyoto, Japan), and major ions were measured using an ion chromatograph (IC, ICS-2100, Dionex, Sunnyvale, CA, USA). The concentrations of trace metallic elements were quantified using inductively coupled plasma mass spectrometry (ICP-MS, Elan DRC-e, PerkinElmer, Waltham, MA, USA). Alkalinity was measured using a digital titrator (Hach, Loveland, CO, USA) and 1.6 N sulfuric acid standard solution. The TDS concentration was measured following the evaporation method at 180 °C after filtering the water samples using a 0.45 μm cellulose acetate membrane filter (Toyo Roshi Kaisha, Ltd., Japan).

### 2.2. Bench- and Pilot-Scale Electrodialysis Systems

**Figure 1:**
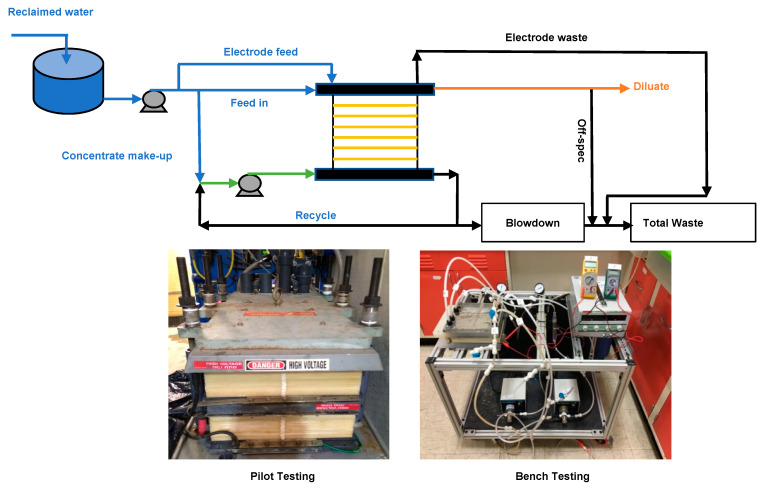
Schematic flow diagram of the electrodialysis systems.

The mobile, trailer-mounted EDR system (AQ3-1-4, Suez Water Technologies & Solutions, Guelph, Ontario, Canada) is a pilot version of a commercial full-scale water treatment plant (Figure 1). The pilot-scale EDR system can generate 3 to 13 gallons per minute (gpm, or 11.3 to 49 L per minute, L/min) product water. The Ionics normal grade IEMs (AR204-SZRA-412 and CR67-HMR-412, Suez Water Technologies & Solutions, Guelph, Ontario, Canada) were used for the bench- and pilot-scale electrodialysis testing. The thickness of the membranes is 0.6 mm for cation-exchange membranes CR67 and 0.5 mm for anion-exchange membranes AR204. Both IEMs have the same water content of 46%. The ion exchange capacity (IEC) of AR204 was higher than the CR67 (2.4 vs. 2.1 meq/g dry membrane), while the area electrical resistance of the AR204 measured in 0.01 N NaCl solution was lower than the CR67 (8 vs. 12 ohm–cm^2^). The patented mesh spacers (Mark IV-2) have a thickness of 0.076 cm and provide flow channel and mixing for each stream in the EDR unit [40]. Platinum plated titanium metal plates were used as electrodes to apply electrical potential to the stack. One hundred cell pairs of CR67 and AR204, with effective area approximately 3200 cm^2^ (3.44 ft^2^) for each membrane, were installed in the EDR stack with 2 electrical stage and 2 hydraulic stage. Each stage had 50 pairs of membranes. An independent voltage source was applied to each electrical stage.

An in-line 10 μm cartridge filter was installed as a pretreatment step before the reclaimed water entering the electrodialysis stack. Feedwater was split into three streams: (I) a stream of feedwater (feed in) was progressively demineralized as desalted water (product water or diluate); (II) a stream of feedwater was used as concentrate make-up to be mixed with concentrate recycled as the inlet concentrate; and (III) a small stream of feedwater (<4%) was diverted into the electrode rinsing stream. A blower was installed to remove the chlorine gas generated in the electrode rinse waste stream. The electrode rinse solution can be replaced by another source of aqueous solution (e.g., Na_2_SO_4_ solution) to avoid the generation of chlorine gas.

The pilot-scale EDR stack reversed electrical polarity automatically every 15 min. The current in the positive polarity mode was slightly smaller than that in the negative polarity mode, which had relatively lower value of electrical conductivity in product water. All the reported data herein were from the water samples obtained during the positive polarity mode, which had similar water quality as in the negative polarity. Within the first minute after polarity change, foulants and scales were flushed off from the IEMs surfaces, and the product water (namely off-spec water) could not meet the required water quality. A real-time conductivity sensor was installed in the diluate stream outlet to monitor the water quality and control a diversion valve to steer the off-spec water into the waste stream when its TDS exceeded the product water quality control level. Both off-spec and electrode rinsing water were diverted into the waste stream, which can be recycled to the feedwater tank for further enhancing water recovery in the EDR system.

Different water recoveries (Equation (2) in Supplemental Information) were achieved by adjusting the flow rates of the concentrate blowdown, feed in, and concentrate make-up. For example, to achieve an overall 50% water recovery, the total inlet feedwater was 14 gpm (53 L/min) to produce 7.5 gpm (28.4 L/min) product water. Considering the water loss as off-spec water (~7% of product water), electrode rinsing waste 0.53 gpm (2 L/min), concentrate make-up stream 5.8 gpm (22.0 L/min), the overall water recovery was 50%. Operating parameters, including applied voltage, current density, pH, electrical conductivity, pressure, and flow rate of each water stream, were recorded by the supervisory control and data acquisition system (SCADA, Allen-Bradley, Milwaukee, WI, USA). The polyphosphate antiscalant (Hypersperse, MDC714, Suez Water Technologies & Solutions) with injection dosage (19 mg/L) calculated by the predictive WATSYS^TM^ program was pumped continuously into the concentrate stream to inhibit scale formation/precipitation on the IEM surface. The WATSYS^TM^ program was developed by Suez Water Technologies & Solutions for designing full-scale EDR systems.

A bench-scale electrodialysis system with the same flow design as the pilot unit was operated independently alongside the pilot-scale EDR system to optimize the desalination performance and scale-up from the bench- to pilot-scale systems. The bench-scale laboratory unit included eleven pieces of CR67 and ten pieces of AR204 membranes, each with 220 cm^2^ (0.24 ft^2^) effective membrane area. The variable-flow micro-pumps (EW-75211-10, Cole-Parmer, Vernon Hills, IL, USA) were chosen to provide desired flow rates and pressure while minimizing pressure fluctuation inside the bench-scale electrodialysis stack. The two electrodialysis systems had the same stack design, configuration of spacers, and IEMs, which made a highly defensible comparison between the bench- and pilot-scale experiments possible. Before each experiment, all new membranes were equilibrated with the reclaimed water for at least 24 h. After the three-month pilot-scale testing, the membranes were removed from the pilot-scale EDR stack for membrane autopsy to identify possible fouling/scaling.

## 3. Results and Discussion

### 3.1. LCD Measurement

Limiting current density (LCD) was studied to evaluate the concentration polarization (CP) in the electrodialysis system. Due to higher ion mobility through membrane matrix than that in solution, concentration polarization, also referred to as concentration gradient, was developed at the interface between membrane and solution. With an increase of the applied voltage, ion concentration on the diluate side would approach zero at the interface, and the current per unit effective membrane area at this point was known as LCD [41]. Water splitting occurs when the current density exceeds the LCD, resulting in reduced desalination efficiency by diverting electric energy to produce H^+^ and OH^−^ from water electrolysis and contributing little to ion transport during desalination [42]. Most electrodialysis and EDR systems are commonly operated at a sub-LCD to achieve high energy efficiency [22], making the LCD an essential parameter in the electrodialysis process.

**Figure 2:**
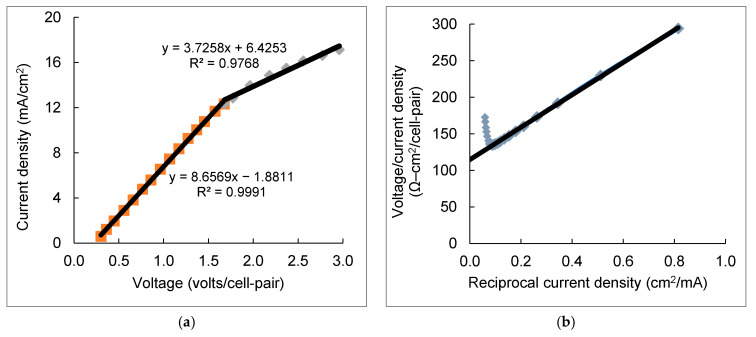

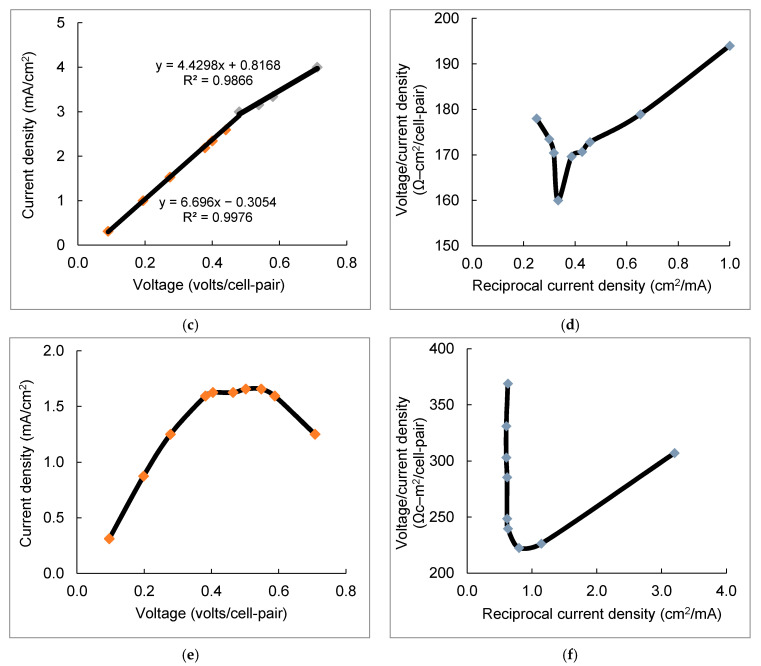
Determination of limiting current density during electrodialysis of reclaimed water. (**a**) and (**b**) V–I and V/I–1/I curves of the bench-scale electrodialysis (ED) system; (**c**) and (**d**) V–I and V/I–1/I curves of the 1^st^ electrical stage in the pilot-scale electrodialysis reversal (EDR) system; (**e**) and (**f**) V–I and V/I–1/I curves of the 2^nd^ electrical stage in the EDR system.

During the bench- and pilot-scale electrodialysis experiments, the applied voltage was increased discretely every ten to fifteen minutes. LCD values were estimated based on the voltage–current response after a steady-state operational condition was reached. Two current–voltage characteristics were adopted to determine the LCD value (Figure 2). The point that the curve slope changes in the voltage–current (V–I) curve indicated the LCD location in Figure 2a,c. Sometimes it is impractical to identify the slope-changing point in the V–I curve, especially during desalination of high salinity water where consistent linearity of V–I relationship could be observed over a broad applied voltage range [43]. Cowan and Brown [44] developed the well-accepted method of plotting V/I against the 1/I curve, where the point with the minimum value of V/I was determined as the location of LCD (Figure 2b,d).

The LCD value of the bench-scale unit was identified to be approximately 12 mA/cm^2^ (Figure 2a). The slope-turning point of the V/I–1/I curve in bench-scale electrodialysis was located at 135 Ω–cm^2^/cell-pair (Figure 2b), in accordance with the observation in Figure 2a. For the 1st electrical stage of the pilot-scale EDR, the LCD was determined to be 3.0 mA/cm^2^ (Figure 2c), while the estimation of the normalized electrical resistance at the LCD was close to 160 Ω–cm^2^/cell-pair (Figure 2d). The ohmic regime below the LCD had a highly linear correlation of current density vs. applied voltage in both the bench- and pilot-scale electrodialysis stack (R^2^ > 0.99), revealing nearly constant electrical resistance during desalination within this current density range. Significant electrical resistance jump was encountered above the LCD point due to the depleting ion concentration at the membrane-solution interface, indicating lower energy efficiency compared to that in the ohmic regime. The data for LCD determination were collected during the positive polarity mode. The LCD measured in the negative polarity exhibited a similar trend but slightly lower by 8% (2.75 mA/cm^2^) than the data measured in the positive polarity mode. The 2nd electrical stage of the pilot-scale EDR exhibited different V–I and V/I–1/I behaviors (Figure 2e,f) compared to the 1^st^ electrical stage. A lower minimum point located at current density 1.25 mA/cm^2^ with higher normalized electrical resistance 220 Ω–cm^2^/cell-pair was detected, due partly to the fact that the inter-stage diluate out, product water of the 1st electrical stage, was the feedwater of the 2nd electrical stage. LCD was reported to be proportional to feedwater salt concentration [43]. The non-linear V–I curve in the 2nd electrical stage (Figure 2e) was mainly caused by the varying electrical resistance associated with the changing salt concentration in its feedwater [45]. Similar trends were observed in our previous experiments of treating brackish groundwater using the same pilot-scale EDR stack at the Kay Bailey Hutchison Desalination Plant in El Paso, Texas [46].

**Figure 3:**
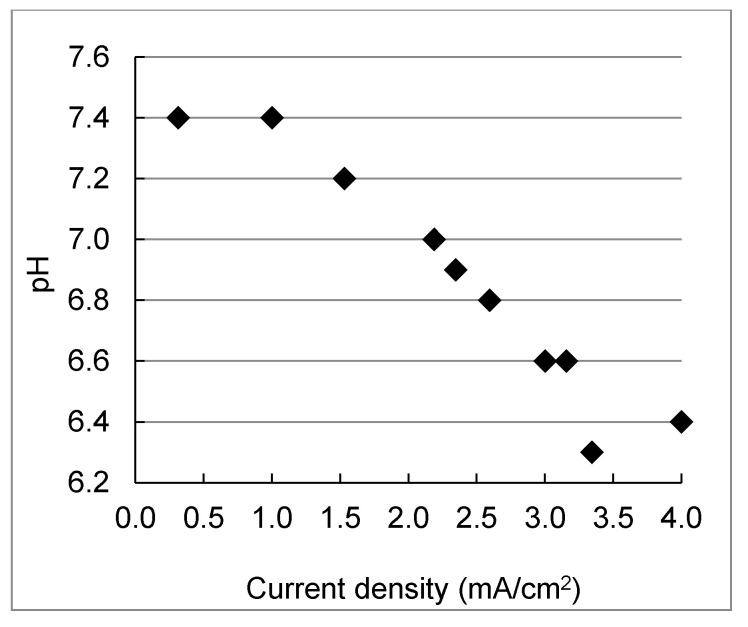
Product water pH as a function of the current density of the pilot-scale EDR system during electrodialysis of the reclaimed water.

For the 1^st^ electrical stage, the pH of the product water decreased from 7.4 (same as the feedwater) to 6.4 with the increase of current density (0.3–4.0 mA/cm^2^) in Figure 3, indicating the water dissociation at higher current density [47], while in the concentrate stream, the pH remained relatively constant at 7.3. The same pH reduction trend was also observed in the negative polarity mode.

### 3.2. Effect of Flow Rate on Desalination Performance

**Figure 4:**
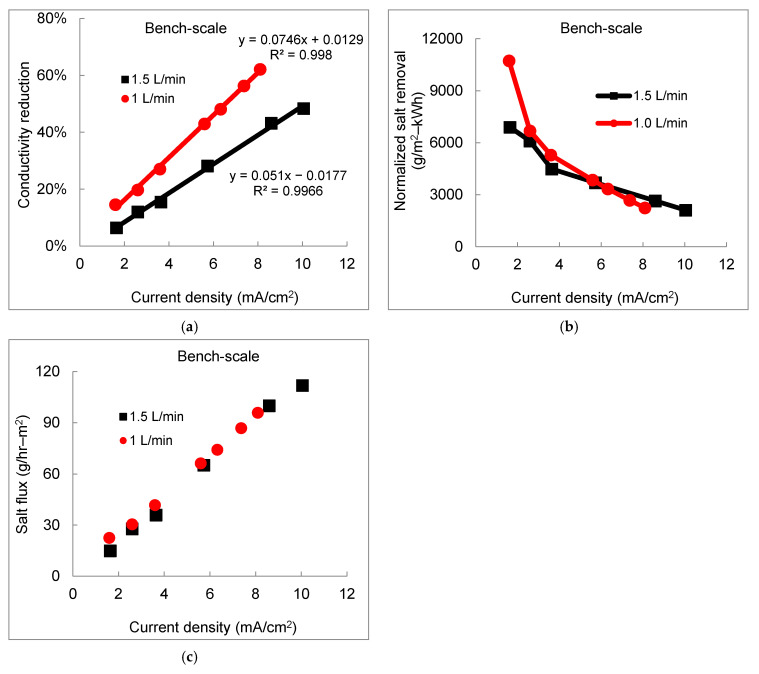
The effect of flow rate on desalination performance in the bench-scale electrodialysis system. (**a**) total dissolved solids (TDS reduction efficiency in terms of conductivity reduction; (**b**) normalized salt removal; and (**c**) salt flux through IEMs.

To study the influence of flow rate on desalination performance, bench-scale experiments were first conducted at a product water flow rate of 1 L/min and 1.5 L/min, corresponding to a linear velocity of 4.1 and 6.1 cm/s, respectively. The percentage of TDS reduction (Equation (1) in Supplemental Information) decreased with the increase of flow rate from 1 L/min and 1.5 L/min (hydraulic residence time 13 s and 9 s, respectively), and higher current density was required to achieve the same conductivity reduction at a higher flow rate (Figure 4a). The difference of TDS removal efficiency at the same current density became prominent from <9% below the current density of 1.5 mA/cm^2^ to >19% above the current density of 8.1 mA/cm^2^. The positive impact of a higher flow rate on salt removal, associated with higher linear velocity, was attributed to the decreased resistance of the boundary layer by suppressing the diffusion layer between solution and membrane surfaces at the side of diluate streams [48]. On the other hand, decreased residence time at a higher flow rate posed a negative effect on salt removal due to lower residence time for ion separation by passing through IEMs within the electrodialysis stack. The overall negative impact of higher flow rate was observed at the current density below approximately 5.6 mA/cm^2^ (Figure 4b), consistent with other observations in bench-scale electrodialysis using normal grade IEMs [46,49,50], which demonstrated the greater influence of residence time over the change of the boundary layer conditions at varying linear velocity.

With increasing current density, the boundary layer became the limiting factor of ion transport through IEMs due to depletion of ions and sharp concentration gradient within the boundary layer. At a current density greater than 6 mA/cm^2^, the significance of positive impact at a higher flow rate prevailed the negative effect of less residence time, resulting in slightly enhanced overall normalized salt removal (Equation (5) in Supplemental Information) at a higher flow rate (Figure 4b). The normalized salt removal exhibited a continuously decreasing trend with increase of the current density at both flow rates, which indicated the higher energy consumption at higher desalination requirement.

To predict the specific salt concentration in product water as a function of current density, the absolute ion transport rate (namely, salt flux, Equation (4) in Supplemental Information) from the diluate stream to the concentrate stream was calculated. Salt flux through IEMs under different flow rates is depicted in Figure 4c. The identical linear correlation between salt flux and current density demonstrated salt flux in electrodialysis is independent of hydrodynamic conditions, including the variation of linear velocity and boundary layer condition. The salt flux is primarily proportional to the current density, the driving force of ion transport [51]. Therefore, the linear variation of the salt flux and electrical charge density can be utilized to predict mass transport under different operating conditions with the same IEMs and spacers installed in the electrodialysis stack.

**Figure 5:**
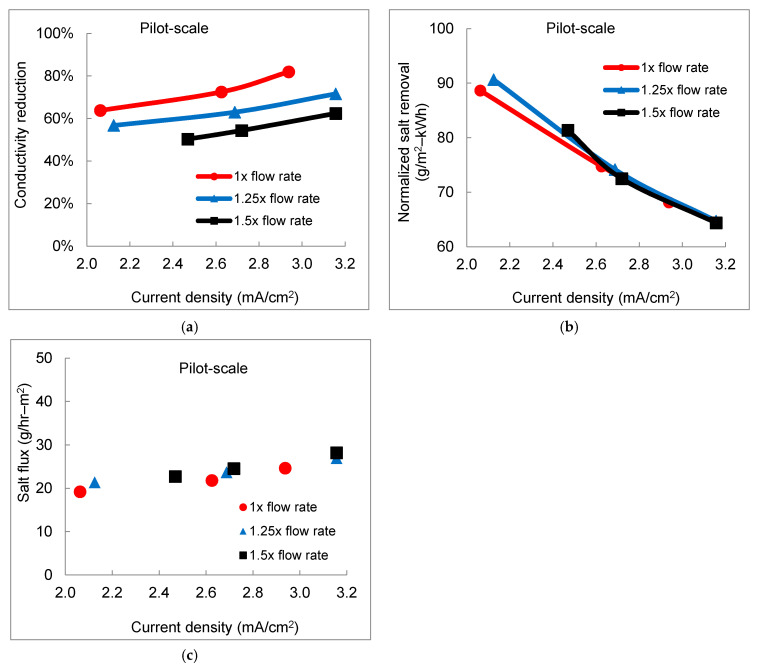
The effect of flow rate on desalination performance in the pilot-scale EDR system. (**a**) TDS reduction efficiency in terms of conductivity reduction; (**b**) normalized salt removal; and (**c**) salt flux through ion exchange membranes (IEMs).

In the pilot-scale testing, a baseline flow rate, namely, 1× flow rate of 7.5 gpm (28.4 L/min product water), was chosen because of the same linear velocity as that in the bench-scale experiments at flow rate 1.5 L/min (a linear velocity of 6.1 cm/s). Moreover, with 1× flow rate (baseline), 1.25× flow rate of 9.4 gpm (35.5 L/min product water), and 1.5× flow rate of 11.3 gpm (42.6 L/min product water), the flow rate conditions were investigated in the pilot-scale EDR system. The percent salt removal efficiency exhibited decreasing trends as the flow rate increased under the same current density (Figure 5a). The normalized salt removal indicated an overall positive impact of a higher flow rate at the starting operating conditions (Figure 5b). Unlike having a short residence time in the bench-scale testing (13 s vs. 9 s), the residence time was 60 s, 48 s, and 40 s corresponding to 1×, 1.25×, and 1.5× flow rate, respectively. Salt ions in the diluate stream had a relatively long enough contact time to transport through IEMs, minimizing the influence of residence time on salt removal. The impact of a higher flow rate with increasing linear velocity demonstrated a dominant positive impact at initial current density tested in the pilot-scale electrodialysis, contrary to the overall negative effect of increased flow rate on desalination performance at low current density in the bench-scale system. Salt flux as a function of current density exhibited a single linear trend, regardless of the different flow rates in the pilot-scale testing (Figure 5c). The trend further confirmed the linear correlation of salt flux as a function of current density.

During electrodialysis of the reclaimed water, the bench-scale system achieved higher normalized salt removal of 6.10 kg salt/m^2^–kWh at the current density of 2.6 mA/cm^2^, while the pilot-scale system only achieved 0.08 kg salt/m^2^–kWh at the current density of approximately 2.6 mA/cm^2^ under the same linear velocity of 6.1 cm/s. The difference of normalized salt removal between the bench- and pilot-scale electrodialysis systems is attributed to the electrodialysis stack configuration and setup. The bench-scale unit has a smaller effective membrane area and a shorted flow path than the pilot system. This short flow path, combined with short hydraulic retention time (HRT), resulted in relatively smaller conductivity change between influents and effluents of diluate and concentrate chambers and reduced water splitting at the end of diluate stream, thus increasing desalination performance. This phenomenon was reported by another study using a different electrodialysis system (PCCell 64 0 02, PCCell GmbH, Germany) with a 64 cm^2^ effective membrane area of the same membrane type but different spacers [52]. A significantly higher normalized salt removal was observed in comparison with this study in treating solution with similar TDS (~1100 mg/L) in bench-scale testing. This result was also confirmed in treating brackish groundwater, using the same bench- and pilot-scale electrodialysis systems as in this study [46].

**Figure 6:**
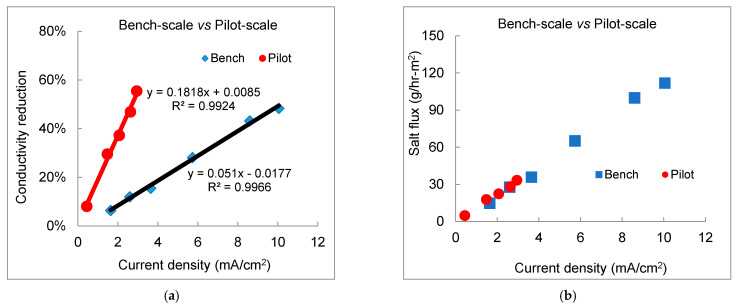
Scale-up of desalination performance from the bench- to pilot-scale electrodialysis of reclaimed water. (**a**) TDS removal in terms of conductivity reduction; (**b**) overall salt flux.

As linearity of salt removal efficiency and salt flux found in both the bench- and pilot-scale experiments, the overall desalination performance from the bench- to pilot-scale electrodialysis was expected to scale-up under the same hydraulic configurations (e.g., linear velocity, spacer pattern, and staging) [46]. With the same linear velocity of 6.1 cm/s, the desalination performance of the bench-scale and the 1^st^ electrical stage in the pilot-scale electrodialysis is evaluated in Figure 6. Generally, both systems exhibited similar trends with increasing current density. High-linear correlations between percent salt removal efficiency and current density (Figure 6a) were observed (R^2^ > 0.99) in the bench-scale and 1^st^ electrical stage of the pilot-scale system. The exhibited overlapping salt flux trends indicated the nearly identical normalized desalting capacity between the bench- and pilot-scale electrodialysis systems with scalable geometry and similar hydrodynamics (Figure 6b). It represented a high similarity in desalination performance, which can be utilized to evaluate the overall desalination of large-scale electrodialysis based on experiments of the scale-down bench-scale system. The desalination efficiency can be expressed as:

Conductivity reduction in pilot-scale system = α × Conductivity reduction in bench-scale unit × (Current Density pilot/Current Density bench) [46].

The ratio α was approximately 3.6 from Figure 6a, matching the ratio (3.4) of the residence time of pilot- over bench-scale experiments (HRT of 30 s over 9 s). The consistency of high similarity between bench- and pilot-scale testing treating reclaimed water (TDS~1150 mg/L, this study) and brackish groundwater (TDS~2700 mg/L, our previous study) presented a reasonable accuracy in simulating pilot-scale electrodialysis system using bench-scale testing results.

### 3.3. Impact of Applied Current Density on Desalination and Ion Separation

Concentration polarization with increased current density has a significant impact on the desalination performance of electrodialysis and EDR systems [53]. To determine the current density that ensures meeting the required standards of target ions (i.e., Na^+^< 110 mg/L, Cl^−^ < 150 mg/L), the pilot-scale EDR system was operated at several current densities for treating the reclaimed water at the baseline flow rate of 7.5 gpm (28.4 L/min product water, or 6.1 cm/s). Table 1 summarizes the corresponding parameters of the 1^st^ and 2nd electrical stages in the EDR pilot-scale system.

With increasing applied voltage and current density, the salt removal efficiency increased, coupling with the increased flux of different ions and reduced salt concentration in the product water. The overall percent salt removal efficiency increased from 45% (Case#1) to 82% (Case#4). Meanwhile, the highest percent TDS reduction efficiency of any single hydraulic stage was below 60% at the four cases tested, within the typical optimal design range of commercial EDR system [54]. The sodium concentration in the product water decreased from 194 mg/L to 126 mg/L from Case#1 to Case#2. The chloride concentration dropped to 101 mg/L in the product water of Case#2, meeting the required goal (<150 mg/L), while the sodium concentration was 97 mg/L in Case#3. Using the correlation of current density and salt concentration in the WATSYS^TM^ program, the goal of sodium concentration 110 mg/L in the product water can be met at the current density of 2.36 mA/cm^2^ for the 1^st^ electrical stage and 1.5 mA/cm^2^ for the 2^nd^ electrical stage. In contrast, chloride concentration in product water was significantly below the requirement (<100 mg/L). Therefore, the pilot-scale EDR system using the normal grade membranes can meet the sodium and chloride water quality goals of treating reclaimed water for golf course irrigation and surface discharge. The normalized salt mass removal exhibited a decreasing trend with the increase of current density, e.g., 0.12 kg salt/m^2^–kWh to 0.07 kg salt/m^2^–kWh from Case#1 to Case#4, revealing reduced energy efficiency when operating at higher current density.

**Figure 7:**
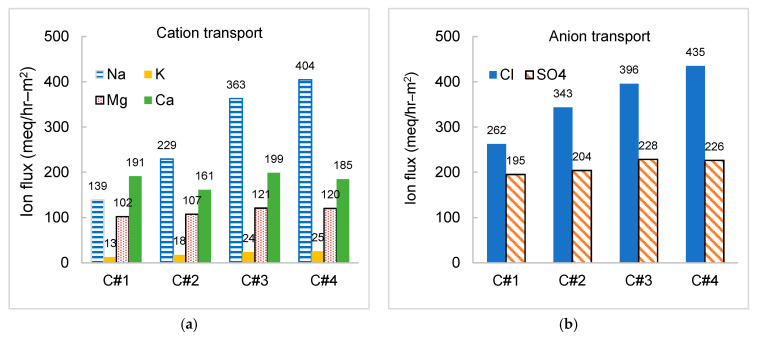
Ion transport in the pilot-scale EDR for treating the reclaimed water at 50% water recovery and baseline flow rates. (**a**) Ion flux of major cations; and (**b**) ion flux of major anions.

The specific ion transport is depicted in Figure 7. In Case#1, the flux of calcium was dominant, reaching 191 meq/hr-m^2^ compared to 139 meq/hr–m^2^ for sodium (Figure 7a), even though the ratio of equivalent concentration of calcium over sodium was less than 0.4 in the feedwater. Selective transport of magnesium over sodium was also noticed, considering the lower ratio of equivalent concentration of magnesium over sodium in the feedwater (approximately 0.2). The selective transport of divalent cations (e.g., Ca^2+^ and Mg^2+^) over monovalent cation (Na^+^) was mainly attributed to the higher electrostatic attraction between the CEMs and the divalent ions with two charges, in comparison to monovalent cation with one charge [46]. For instance, the percent removal efficiency of calcium and magnesium reached 84% and 83% at Case#1 with low current density, respectively, while removal efficiency of 24% and 39% were recorded for sodium and potassium, respectively. Enhanced transport of divalent anion (SO_4_^2^^−^) was also detected (195 meq/hr–m^2^) over monovalent anion (262 meq/hr–m^2^ for Cl^−^) in Figure 7b, in respective to less than 0.5 of equivalent concentration ratio of sulfate over chloride in the feedwater. With increasing current density, the increase of salt removal was mostly carried out by the transport of monovalent ions. The flux of monovalent ions increased linearly with the increase of current density, while divalent ions displayed a non-linearity trend.

Selective separation of divalent ions vs. monovalent ions was studied based on Na selectivity for cations and Cl selectivity for anions, defined as relative transport number (RTN, Equation (6) in Supplemental Information). The RTN calculation is based on the widely-adopted equation developed by Sata et al. [55]. Na^+^ was specified as the reference cation, and Cl^−^ was chosen to be the reference anion. The RTN is measured as the amount of a given ion removed (in meq) divided by its arithmetic average equivalent concentration of initial and final concentration in the diluate streams, as opposed to the amount of reference ion removed (in meq) divided by the average arithmetic equivalent the reference ion concentration of initial and final concentration.

**Figure 8:**
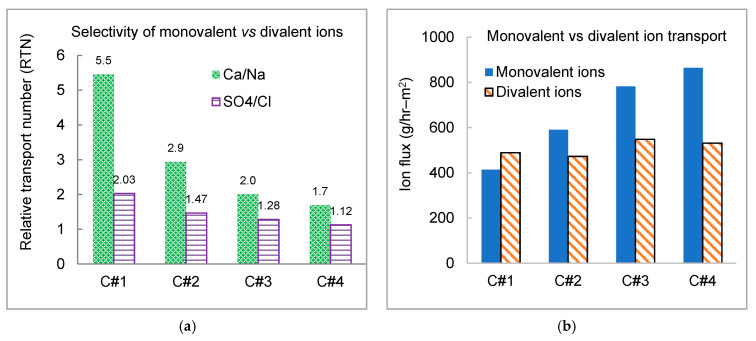
Selectivity of monovalent vs. divalent ion (**a**) relative transport number of calcium over sodium and sulfate over chloride; and (**b**) ion flux of monovalent over divalent ions.

The trend of the RTN value of calcium was similar to that of magnesium, indicating similar transport behavior for the major divalent cations. Divalent ions were selectively transported over monovalent ions (RTN > 1) in the entire current density range tested. The elevated removal of divalent ions was pronounced at low current density, i.e., the maximum RTN value of 5.5 for calcium (Figure 8a). The downtrend of RTN for both divalent cation and anion was attributed to the sharp concentration gradient in the boundary layer as current density increased, enhancing the transport of monovalent ions in the boundary layer because of higher diffusivity of monovalent over divalent ions [56] and the downshifted degree of increment for divalent removal with the increase of current density (Figure 7). Furthermore, the total flux of monovalent ions increased linearly with the increase of current density, while the overall flux of divalent ions fluctuated in a relatively narrow range around 500 meq/hr–m^2^ (Figure 8b).

Comparison between the measured individual ion concentration data and the projection results using the WATSYS^TM^ program was investigated (Figure S1 in Supplemental Information). The WATSYS^TM^ program projected well the overall salt removal in terms of conductivity reduction and pH change. However, there were discrepancies between the measured ion concentrations and the projection results. The program also projected fairly well the transport of monovalent ions, while for divalent ions (e.g., Ca^2+^, Mg^2+^, and SO_4_^2−^), the differences between projected and measured data were in the range of 10–40%. The monovalent ions demonstrated a fairly linear correlation between current density and ion flux, while non-linearity was noticed in divalent ions separation (Figure 8b). The nonlinear trend of divalent ions might contribute to the discrepancies between the measured and the WATSYS^TM^ projected removal data.

A low concentration of organic matter existed in the reclaimed water, measured as TOC of 7.7 ± 0.2 mg/L. The TOC concentration in product water ranged from 7.2 mg/L to 7.6 mg/L in the bench-scale electrodialysis system. One product water from Case#1 was measured as 5.03 mg/L of TOC. It was reported that organic matter in the reclaimed water is mostly neutral substances, which would not migrate under electric field [57]. Therefore, organic removal was not further discussed in this study because there was no significant decrease of TOC in product water using electrodialysis.

### 3.4. Effect of Water Recovery

**Figure 9:**
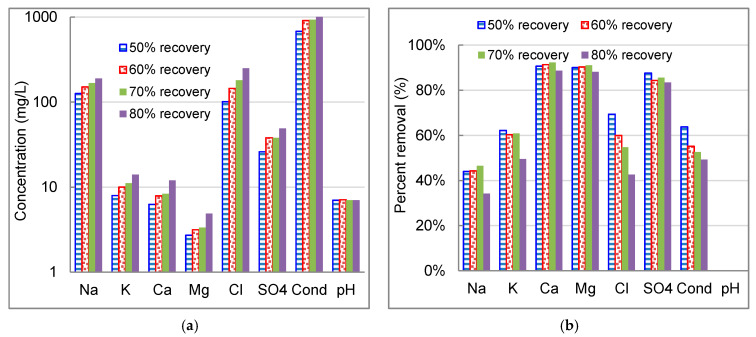
Salt rejection as a function of water recovery in treating reclaimed water (**a**) water quality in product water; (**b**) percentage removal of conductivity and major ions.

The salt reduction decreased with the increase of water recovery in the pilot-scale EDR system due to a higher concentration gradient between the diluate and concentrate streams. The variation of conductivity reduction was less than 15% when the water recovery increased from 50% to 80%. The concentration of individual ion increased in product water as the water recovery increased (Figure 9a). The pH of product waters at different water recovery was closely matching (pH 7.0–7.1). The variations of percent removal of the major cations were within 2% as water recovery ranging from 50% to 70%, except a sharp drop at water recovery of 80% (Figure 9b). The transport of chloride was relatively more sensitive to water recovery compared to other major ions. The highest equivalent concentration of chloride in concentrate might restrain the chloride transport from the diluate to concentrate stream.

**Figure 10:**
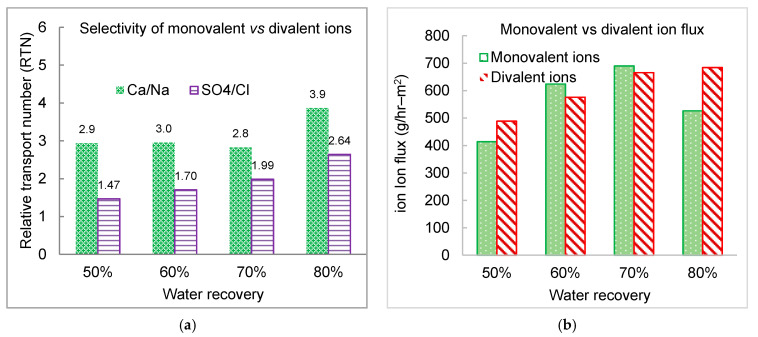
Selectivity of monovalent vs. divalent ion as a function of water recovery. (**a**) Relative transport number; and (**b**) ion flux of monovalent over divalent ions.

The selective separation of divalent ions vs. monovalent ions is presented in Figure 10. For cations, the RTN value (2.8–3.0) was relatively stable when the water recovery increased from 50% to 70%, while a sharp increase to 3.9 (~40% increase) at water recovery of 80%. Stronger back diffusion potential of monovalent cations from the concentrate streams contributed to the enhanced divalent cation selectivity at higher water recovery. However, the RTN value of sulfate steadily increased from 1.47 to 1.99 as water recovery increased from 50% to 70%, then jumped to 2.64 at water recovery of 80%.

After finishing all the experiments, minor inorganic scaling was spotted on the CEM surface, while no scalants were observed on the AEM surface by comparing the used and pristine membranes.

### 3.5. Techno-Economic Analysis

Based on the testing results, alternative treatment configurations were developed in comparison to the existing UF/RO system in Scottsdale Water Campus as baseline. The blending analysis (Table 2) and cost comparison (Table 3) were presented for a one million gallon per day (mgd, or 3785 m^3^/d) feed water flow on a realistic and common basis. Cost evaluation was performed in accordance with the Association for the Advancement of Cost Engineering (AACE) [58]. A detailed cost comparison was developed to evaluate the total capital cost, annual operation and maintenance (O&M) cost, and 20-year life cycle cost for the proposed treatment alternatives (Detailed assumptions in Supplemental Information). Table 2 and Table 3 summarize the calculated costs and system configuration and performance for the UF/RO system as well as the 2-stage EDR and 4-stage EDR design based on WATSYS^TM^ simulation and pilot testing results.

The estimated treatment costs of the electrodialysis processes were proven lower than using UF/RO to treat reclaimed water for golf course irrigation. The economic evaluation based on the WATSYS^TM^ data revealed a more than 21% total cost reduction while satisfying the product water quality requirements (shown in Table 2 and Table 3). The total life cycle cost was reduced by approximately 19% using 2-stage EDR system based on the testing data. The chloride concentration was below the target goal of 150 mg/L. However, the sodium concentration cannot meet the operating goal of 110 mg/L, though very close to the contractual goal of 125 mg/L based on the pilot-scale testing results. Therefore a 4-stage EDR system was designed to meet both water quality requirements for sodium of 110 mg/L and chloride of 150 mg/L as well as to reduce the costs for treating reclaimed water for golf course irrigation. For a 4-stage EDR system, the unit O&M cost would be reduced to $0.83/kgal ($0.22/m^3^) from $1.09/kgal ($0.29/m^3^) in the baseline condition. The total life cycle cost was reduced to $11,900,000 and $12,400,000 in the WATSYS^TM^ simulation and evaluation based on the testing data, respectively, in comparison to $12,800,000 in the UF/RO baseline.

In addition to the reduced treatment costs, the EDR system has the advantage of achieving high water recovery: 90% unit water recovery and 92–93% overall water recovery considering water blending, while the UF/RO system could only achieve 85% unit water recovery and 88% overall water recovery. The TDS concentration of the product water of the EDR system is estimated better than the RO system: 433 mg/L for the 4-stage EDR system vs. 530 mg/L for UF/RO.

In summary, the techno-economic modeling indicates that using electrodialysis for treating reclaimed water is more cost-effective and can recover more water than the RO process.

## 4. Conclusions

In this study, bench- and pilot-scale electrodialysis experiments were conducted to assess the technical feasibility and economic viability of reclaimed water desalination. The study elucidated the effects of various operating conditions, such as flow rate, HRT, applied current density, staging, and water recovery, on overall salt removal and monovalent permselectivity during reclaimed water treatment using electrodialysis.

The overall negative impact of a higher flow rate was observed at the current density below 5.6 mA/cm^2^ for the bench-scale experiments. In comparison, the impact of a higher flow rate with increased linear velocity demonstrated a dominant positive impact at the initial current density tested in the pilot-scale electrodialysis. The linear trend of salt removal efficiency and salt flux was observed in both bench- and pilot-scale experiments, with overlapping linear trends of salt flux and scalable desalination efficiency (matching the ratio of residence time) between the bench- and pilot-scale electrodialysis systems. Such linear correlation of the salt flux and electrical charge density can be utilized to predict solute mass transport under different operating conditions with the same IEMs and spacers installed in the electrodialysis stack.

The pilot-scale EDR system using the normal grade membranes demonstrated the technical feasibility of treating reclaimed water for golf course irrigation and surface discharge. An increase in the applied voltage enhanced the removal efficiency of ion separation and reduced the salt content in the product water. The target goal of sodium concentration 110 mg/L in the product water can be met at the current density of 2.36 mA/cm^2^ for the 1^st^ electrical stage and 1.5 mA/cm^2^ for the 2^nd^ electrical stage, while chloride concentration in product water was significantly below the requirement (<100 mg/L). Monovalent-selectivity in terms of equivalent removal of divalent ions (Ca^2+^, Mg^2+^, and SO_4_^2−^) over monovalent ions (Na^+^, Cl^−^) was affected by both current density and water recovery. The salt rejection decreased in the pilot-scale EDR system at higher water recovery, due to a higher concentration gradient between the diluate and concentrate streams. No significant scaling/fouling on the IEM surface in the pilot-scale electrodialysis system was observed throughout the three-month continuous testing period.

Total cost reduction can be achieved in electrodialysis, compared to the existing treatment option. The unit O&M cost would be reduced to $0.83/kgal ($0.22/m^3^) from $1.09/kgal ($0.29/m^3^) in the baseline condition, along with reduced total life cycle cost. The water recovery of the EDR system is higher than the UF/RO, with 90% unit water recovery and 92–93% overall water recovery. In conclusion, the electrodialysis demonstrated promising technical feasibility and economic viability for treating reclaimed water.

## Figures and Tables

**Table 1:** Summary of operational parameters and data in different cases.

Parameters	Case#1(C#1)		Case#2(C#2)		Case#3(C#3)		Case#4(C#4)	
Electrical Stage	1^st^	2^nd^	1^st^	2^nd^	1^st^	2^nd^	1^st^	2^nd^
Voltage, (V)	14	13	19	19	23	21	26	24
Current, (A)	4.7	3.6	6.7	4.8	8.3	4.8	9.5	4.9
Current Density, (mA/cm)^2^	1.5	1.1	2.1	1.5	2.6	1.5	3.0	1.5
Salt Removal, (%)	29.6%	22.3%	37.3%	42.2%	46.9%	48.1%	55.5%	59.4%
Salt Removal Rate, (g/hr)	571	302	719	510	904	492	1070	509
Energy Consumption, (kWh)	65.8	46.8	127.3	91.2	193.2	98.7	244.4	117.6
Energy Consumption, (kWh/kg salt)	0.12	0.15	0.18	0.18	0.21	0.20	0.23	0.23
Na^+^, (mg/L)	244	194	204	126	191	97	169	75
Cl^−^, (mg/L)	279	205	204	101	171	72	147	50

**Table 2:** Blending analysis comparison between EDR and baseline.

Parameters	Baseline (UF/RO)	2-Stage EDR		4-Stage EDR	
		EDR (WATSYS)	EDR (Testing)	EDR (WATSYS)	EDR (Testing)
Feed Water Flow (mgd)	1	1	1	1	1
Feed Water Na (mg/L)	235	235	235	235	235
% Flow Treated	60.5%	69.0%	100.0%	69.0%	78.0%
Overall Recovery	88%	93%	92%	93%	92%
Unit Recovery	85%	90%	90%	90%	90%
Blended Water Flow (mgd)	0.88	0.93	0.92	0.93	0.92
Product Water Na (mg/L)	110	110	129	110	110
Product TDS (mg/L)	530	522	489	522	433
Concentrate Flow (gpm)	60	48	69	48	54
Concentrate TDS (mg/L)	7530	9662	7130	9662	9662
Concentrate Na (mg/L)	1524	2136	1715	1927	1715
Number of Product Line	-	7	8	7	6
Number of Stages	-	2	2	4	4

**Table 3:** Cost comparison between EDR and baseline.

Parameters	Baseline (UF/RO)	2-Stage EDR		4-Stage EDR	
		EDR (WATSYS)	EDR (Testing)	EDR (WATSYS)	EDR (Testing)
UF	$599,000	$-	$-	$-	$-
Residuals Handling	$765,000	$-	$-	$-	$-
RO	$889,000	$-	$-	$-	$-
EDR	$ -	$1,820,000	$2,080,000	$2,520,000	$2,940,000
Building	$652,000	$435,000	$435,000	$435,000	$435,000
Civil Site Works (5%)	$146,000	$113,000	$126,000	$148,000	$169,000
Electrical and I&C (25%)	$726,000	$564,000	$629,000	$739,000	$844,000
Contingency (30%)	$1,189,000	$924,000	$1,030,000	$1,210,000	$1,382,000
General Conditions: Mobilization & Demobilization (5%)	$189,000	$147,000	$164,000	$193,000	$220,000
Engineering, Administration, and Legal (18%)	$928,000	$721,000	$804,000	$944,000	$1,078,000
Total Capital Costs ($)	6,081,000	4,721,000	5,266,000	6,187,000	7,066,000
Unit Capital Costs ($/gpd)	6.88	5.05	5.72	6.64	7.68
Total Power Cost ($/year)	84,000	28,000	20,000	44,000	28,000
Total Chemical Cost ($/year)	69,000	67,000	67,000	67,000	67,000
Total Labor Cost ($/year)	126,000	126,000	126,000	126,000	126,000
Total Replacement Cost ($/year)	17,000	14,000	13,000	14,000	13,000
Contingency (20%)	59,000	47,000	45,000	50,000	47,000
Total O&M Costs	353,000	280,000	269,000	299,000	278,000
Unit O&M Costs ($/kgal)	1.09	0.82	0.80	0.88	0.83
Total Life Cycle Costs ($)	12,800,000	10,100,000	10,400,000	11,900,000	12,400,000

## Data Availability

Detailed experimental data can be provided upon reasonable request.

## Supplementary Materials

The following are available online at https://www.mdpi.com/article/10.3390/membranes11050333/s1. Figure S1. Desalination performance as a function of current density in terms of ion concentration, ion removal, conductivity, conductivity reduction, and pH change. Reference [58] is cited in supplementary material.
