# Supplementary material for: Pilot Demonstration of Reclaiming Municipal Wastewater for Irrigation Using Electrodialysis Reversal: Effect of Operational Parameters on Water Quality
Source: Membranes (Basel). 2021 Apr 30;11(5):333. doi: 10.3390/membranes11050333 (PMC8147136; doi:10.3390/membranes11050333)
Supplement: Supplementary file 1 [file membranes-11-00333-s001.zip › membranes-1196665-supplementary.pdf]

membranes

Supplementary Materials: Pilot demonstration of reclaiming municipal wastewater for irrigation using electrodialysis: effect of operational parameters on water quality

Xuesong Xu [1], Qun He [2], Guanyu Ma [1], Huiyao Wang [1], Nagamany Nirmalakhandan [1] and Pei Xu [1,*]

[1] Department of Civil Engineering, New Mexico State University, Las Cruces, NM 88003, USA; xuesong@nmsu.edu (X.X.); gyma@nmsu.edu (G.M.); huiyao@nmsu.edu (H.W.); nkhandan@nmsu.edu (N.N.)
[2] Carollo Engineers, City of Phoenix, AZ 85034, USA; che@carollo.com
* Correspondence: pxu@nmsu.edu; Tel.: 1-575-646-5870

Citation: Xu, X.; He, Q.; Ma, G.; Wang, H.; Nirmalakhandan, N.; Xu, P. Pilot Demonstration of Reclaiming Municipal Wastewater for Irrigation Using Electrodialysis Reversal: Effect of Operational Parameters on Water Quality. *Membranes* **2021**, *11*, 333. https://doi.org/10.3390/membranes11050333

Academic Editor: Xue Jin

Received: 9 April 2021
Accepted: 28 April 2021
Published: 30 April 2021

Publisher's Note: MDPI stays neutral with regard to jurisdictional claims in published maps and institutional affiliations.

Copyright: © 2021 by the authors. Licensee MDPI, Basel, Switzerland. This article is an open access article distributed under the terms and conditions of the Creative Commons Attribution (CC BY) license (http://creativecommons.org/licenses/by/4.0/).

SUMMARY

Number of pages: 5
Number of Figures: 1

The desalination performance was evaluated in terms of the conductivity of the diluate, percent conductivity reduction, removal of different ions, water recovery, salt flux, and normalized salt removal.

Salt removal in terms of conductivity reduction is defined as

$$\text{Percent conductivity reduction (\%)} = \left(1 - \frac{EC_d}{EC_f}\right) \times 100\%, \quad (1)$$

where EC_d and EC_f are electrical conductivities (μS/cm) in the diluate and the feed, respectively.

The water recovery is the percentage of feed water flow that becomes diluate and is given by

$$\text{Water Recovery} = \frac{Q_d}{Q_f} \times 100\%, \quad (2)$$

where Q_d and Q_f are flow rates (gpm) of the diluate and the feed, respectively.

The current density is defined as

$$\text{Current Density} = \frac{I}{S_1}, \quad (3)$$

where I is the current (amperage) and S_1 is the effective membrane surface area (3,200 cm^2).

The salt flux is defined as

$$\text{Salt flux} = \frac{(C_f - C_d) \times Q_d}{S}, \quad (4)$$

where C_d and C_f are ion concentrations (mg/L) of the diluate and the feed; S is the total membrane surface area of the stack.

The normalized salt removal is given as

$$\text{Normalized salt removal} = \frac{(C_f - C_d) \times Q_d}{S \times I \times V}, \quad (5)$$

where V is the applied voltage to the electrodialysis stack.

The relative transport number (RTN) is calculated as

$$\text{RTN} = \frac{(t_i / t_{ref})}{(C_i / C_{ref})}, \quad (6)$$

where t_i and t_{ref} are the transport number of a specific ion (i.e., Ca^{2+} or SO_4^{2-}) and reference ion (Na^+ for cation or Cl^- for anion) through the membranes, respectively; C_i and C_{ref} are the arithmetic average equivalent concentration of initial and final concentration of a specific ion (i.e., Ca^{2+} or SO_4^{2-}) and reference ion (Na^+ for cation or Cl^- for anion) in the diluate streams, respectively.

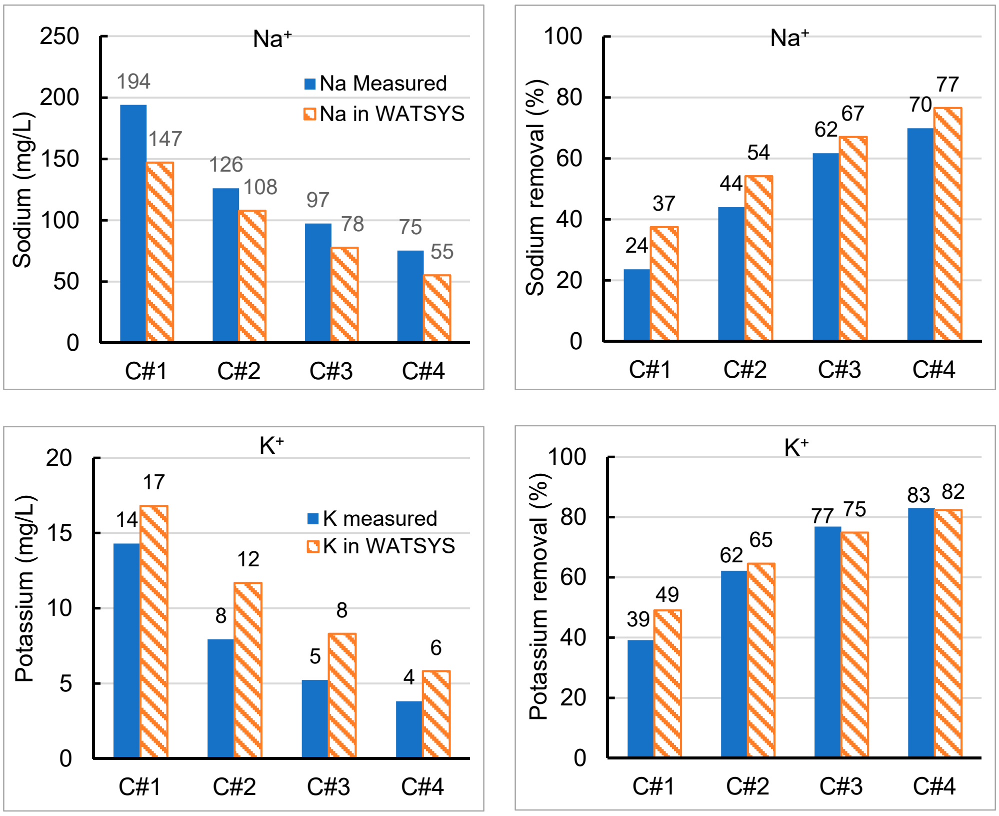

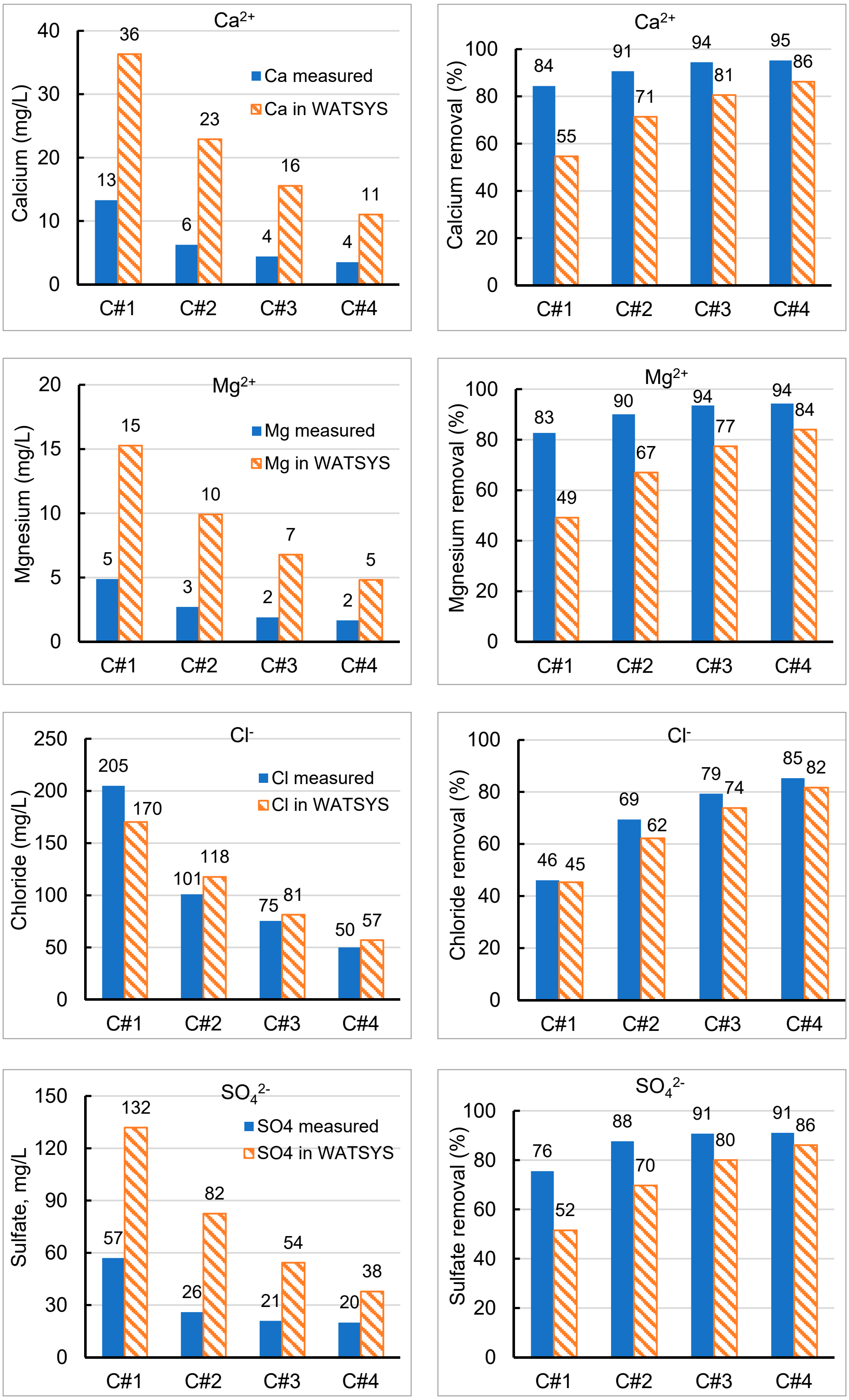
Ca2+
Calcium (mg/L)
Ca measured
Ca in WATSYS
36 23 16 11
13 6 4 4
C#1 C#2 C#3 C#4
Calcium removal (%)
84 91 94 95
55 71 81 86
Mg2+
Mgnesium (mg/L)
Mg measured
Mg in WATSYS
15 10 7 5
5 3 2 2
Mgnesium removal (%)
83 90 94 94
49 67 77 84
Cl-
Chloride (mg/L)
Cl measured
Cl in WATSYS
205 101 75 50
170 118 81 57
Chloride removal (%)
46 69 79 85
45 62 74 82
SO42-
Sulfate, mg/L
SO4 measured
SO4 in WATSYS
57 26 21 20
132 82 54 38
Sulfate removal (%)
76 88 91 91
52 70 80 86

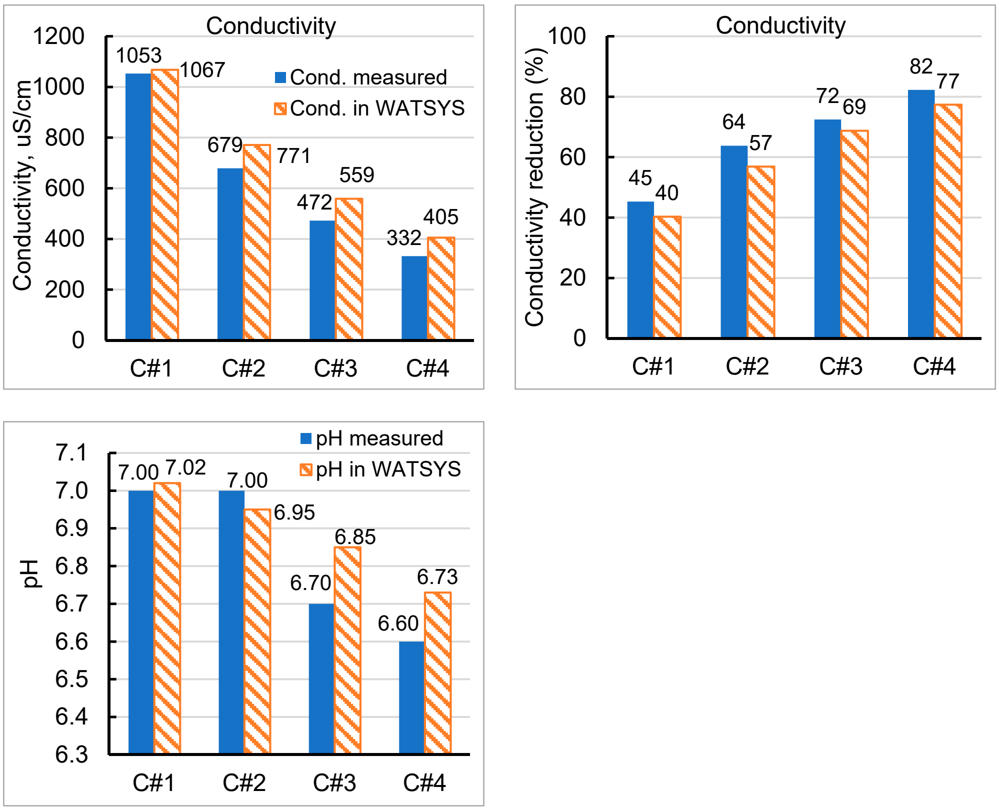

Figure S1. Desalination performance as a function of current density in terms of ion concentration, ion removal, conductivity, conductivity reduction, and pH change.

Based on the testing results, alternative treatment configuration was developed, in comparison to the existing baseline in the Scottsdale Water Campus (UF+RO). The blending analysis and cost comparison were presented on a realistic and common basis using the WATSYS™ and Blue Plan-it™ Decision Support System (BPI). WATSYS™ was developed by Suez Water Technologies & Solutions for designing and maintaining EDR systems. In order to produce detailed evaluations of multiple process options, The BPI developed by Carollo Engineers was utilized to simulate the performance of multiple alternatives (https://www.carollo.com/innovation/blue-plan-it). BPI is a tool to manage complex, interconnected treatment and conveyance systems. This innovative planning tool is a water, salt, and energy balance model that simulates the fresh and brackish water sources flow routing, treatment, distribution, and the associated energy demands and costs, which is particularly suitable in the field of salinity management.

Cost evaluation was performed in accordance with the Association for the Advancement of Cost Engineering (AACE) [1]. A detailed cost comparison was developed to evaluate the total capital cost, annual operation and maintenance cost, and the total 20-year life cycle cost for the proposed treatment alternatives. Major assumptions include

- EDR costs are based on a quotation from the EDR equipment manufacturer in conjunction with the pilot-scale experiment data;
- MF/UF and RO system costs are based on results from Carollo Engineers' previous bid projects utilizing same equipment;
- The Process/Electrical/Control Building assumes a single building with an electrical room and a control room and assumes HVAC and plumbing. The building cost was estimated at $250/sf based on Carollo Engineers' recent projects in Arizona and California;

- The Civil Site Work line item includes excavation and backfill, general site preparation and finishing, with estimation of 5% of the capital costs;
- The Electrical and Instrumentation line item assumes 25% of equipment costs;
- The General line item assumes 5% of the total capital cost for mobilization and demobilization, temporary facilities, startup, testing, and commissioning;
- The CCI number for December 2016 is 10531 (20 City Average) and was used to escalate previous cost references;
- Chemical costs were estimated based on quotes received from local chemical suppliers supplemented by Carollo Engineers' recent reference projects;
- Unit power costs were assumed at $0.08/kWh.

The above assumptions are representative of the current water treatment industry construction pricing in the southwestern United States. Additionally, the costs for contingency, contractor overhead, and profit and engineering are based on industry standards for a project of this nature. Furthermore, 2-stage and 4-stage EDR systems were evaluated in comparison to the existing treatment (UF+RO), respectively. The evaluation was conducted in both WATSYS™ (based on predictive results) and Blue Plan-it™ (based on testing results), independently.

References

1. Christensen, P., Dysert, L.R., Bates, J., Burton, D., Creese, R.C. and Hollmann, J. *Cost Estimate Classification System—As Applied in Engineering, Procurement, and Construction for the Process Industries*; AACE, Inc: Morgantown, WV, USA, 2011.
